# MOF-Derived CoSe_2_@N-Doped Carbon Matrix Confined in Hollow Mesoporous Carbon Nanospheres as High-Performance Anodes for Potassium-Ion Batteries

**DOI:** 10.1007/s40820-020-00539-6

**Published:** 2020-10-27

**Authors:** Su Hyun Yang, Seung-Keun Park, Yun Chan Kang

**Affiliations:** 1 Department of Materials Science and Engineering, Korea University, Anam-Dong, Seongbuk-Gu, Seoul, 136-713 Republic of Korea; 2 Department of Chemical Engineering, Kongju National University, 1223-24 Cheonan-daero, Seobuk-gu, Cheonan, 31080 Republic of Korea

**Keywords:** Metal–organic frameworks, Hollow mesoporous carbon nanospheres, Potassium-ion batteries, Cobalt selenides, Electrode materials

## Abstract

In this work, a novel vacuum-assisted strategy is proposed to homogenously form Metal–organic frameworks within hollow mesoporous carbon nanospheres (HMCSs) via a solid-state reaction. The method is applied to synthesize an ultrafine CoSe_2_ nanocrystal@N-doped carbon matrix confined within HMCSs (denoted as CoSe_2_@NC/HMCS) for use as advanced anodes in high-performance potassium-ion batteries (KIBs). The approach involves a solvent-free thermal treatment to form a Co-based zeolitic imidazolate framework (ZIF-67) within the HMCS templates under vacuum conditions and the subsequent selenization. Thermal treatment under vacuum facilitates the infiltration of the cobalt precursor and organic linker into the HMCS and simultaneously transforms them into stable ZIF-67 particles without any solvents. During the subsequent selenization process, the “dual confinement system”, composed of both the N-doped carbon matrix derived from the organic linker and the small-sized pores of HMCS, can effectively suppress the overgrowth of CoSe_2_ nanocrystals. Thus, the resulting uniquely structured composite exhibits a stable cycling performance (442 mAh g^−1^ at 0.1 A g^−1^ after 120 cycles) and excellent rate capability (263 mAh g^−1^ at 2.0 A g^−1^) as the anode material for KIBs.

**Electronic supplementary material:**

The online version of this article (10.1007/s40820-020-00539-6) contains supplementary material, which is available to authorized users.

## Highlights

- A novel vacuum-assisted strategy is proposed to form N-doped carbon-encapsulated CoSe_2_ nanocrystals within hollow mesoporous carbon nanospheres (CoSe_2_@NC/HMCS) via a solid-state reaction.
- The “dual confinement” by both the N-doped carbon matrix derived from 2-methylimidazole and the small-sized pores of the hollow mesoporous carbon nanospheres can effectively prevent the overgrowth of CoSe_2_ nanocrystals.
- CoSe_2_@NC/HMCS exhibits an excellent electrochemical performance as the anode material for KIBs in terms of cycling stability and rate capability.

**Electronic supplementary material:**

The online version of this article (10.1007/s40820-020-00539-6) contains supplementary material, which is available to authorized users.

## Graphical abstract

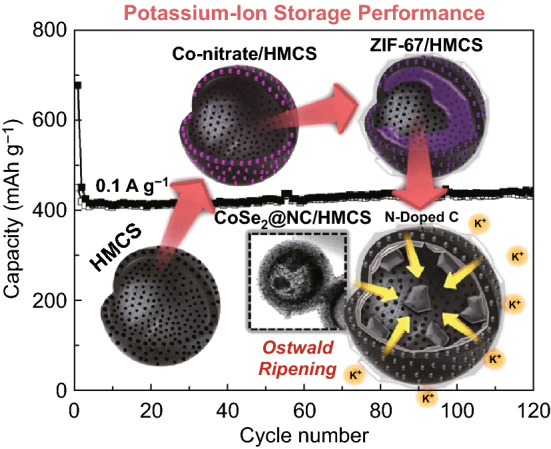

## Introduction

Potassium-ion batteries (KIBs) have recently attracted considerable attention for their high energy densities and the abundant availability of potassium as a raw material [1–3]. Thus, KIBs are being positioned as an alternative energy storage system to lithium-ion batteries (LIBs), which are currently the dominant power sources for electric vehicles and portable electronic devices. Nevertheless, the large radius of K^+^ ions (1.38 Å) leads to a sluggish ion diffusion rate and large volume expansion of the electrode materials during repeated charge–discharge processes, which can hinder KIB commercialization [4, 5]. Therefore, the major challenge for KIBs resides in designing novel electrode materials with improved electrochemical performances, including high specific capacity, long cycling life, and excellent rate capability.

As emerging anode materials for KIBs, transition metal selenides have been intensively studied in consideration of their high specific theoretical capacities and narrow bandgap semiconductor properties [6, 7]. However, two major obstacles still require to be overcome: their large volume expansion upon potassiation and their low electrical conductivity. Thus, to enhance the potassium storage performance of transition metal selenides, many studies have proposed various strategies such as designing unique nanostructured materials and combining them with conductive carbonaceous materials [8–11].

Metal–organic frameworks (MOFs), consisting of metal cation nodes and organic struts, have been considered as ideal templates to synthesize diverse electrode materials owing to their tunable compositions and structures [12, 13]. In particular, upon pyrolysis under inert conditions, the metal cations contained in the MOFs can transform into metallic nanoparticles, which are simultaneously enclosed by the carbon matrix, resulting from the carbonization of the organic struts. By exploiting these features and controlling the atmospheric environment, researchers have obtained various metal compounds such as oxides, sulfides, and selenides embedded in the carbon matrix from MOF templates, and these compounds have been extensively applied as electrode materials for Li- and Na-ion batteries [14–17]. However, only a few studies have applied MOF-based composite to KIBs, and the resulting materials have not thus far afforded a satisfactory electrochemical performance [18–20].

Meanwhile, hollow or porous carbon templates are considered desirable substrates to endow various nanomaterials with structural robustness and electrical conductivity [21, 22]. Thus, several recent studies have suggested confining MOFs within these carbon templates to obtain enhanced structural stability and electrochemical properties [23–25]. However, although most of these studies used a well-known liquid-phase process for synthesizing MOFs, it is difficult to confine the MOF nanoparticles within the carbon templates with this method. As a result, some particles may be individually formed outside the templates, which can degrade the electrochemical performance. Therefore, the current challenge is to develop a new strategy for uniform MOF formation within the carbon templates to enhance the potassium-ion storage properties.

In this study, for the first time to the best of our knowledge, we propose a novel vacuum-assisted strategy to homogenously form MOFs within hollow mesoporous carbon nanospheres (HMCSs) via a solid-state reaction. Applying this method, we successfully synthesize an ultrafine CoSe_2_-nanocrystal@N-doped carbon matrix confined within HMCSs (denoted as CoSe_2_@NC/HMCS) for application as advanced anodes in high-performance KIBs. This approach involves a solvent-free thermal treatment to form a Co-based zeolitic imidazolate framework (ZIF-67) within HMCS templates under vacuum conditions, followed by selenization. The thermal treatment under vacuum facilitates both the infiltration of the cobalt precursor and 2-methylimidazole (2-MIM) into the HMCS and their simultaneous transformation into stable ZIF-67 particles without requiring any solvents. During the subsequent selenization process under a H_2_/Ar atmosphere, the “dual confinement” by both the N-doped carbon matrix derived from 2-MIM and the small-sized pores of the HMCS can effectively prevent the overgrowth of CoSe_2_ nanocrystals. Consequently, tiny CoSe_2_ nanocrystals embedded in the N-doped carbon matrix are homogeneously distributed within the HMCSs. The resulting uniquely structured composite provides a conductive pathway for electrons and enable fast ion diffusion and easy accessibility of the electrolyte through ample pores. Moreover, the volume expansion of CoSe_2_ is effectively suppressed during the electrochemical reaction. Thus, CoSe_2_@NC/HMCS exhibits an excellent electrochemical performance as the anode material for KIBs in terms of cycling stability and rate capability. We also investigate the charge–discharge mechanism of the electrode by applying various analytic tools.

## Experimental Section

The CoSe_2_@NC/HMCS composite was prepared through a two-step process composed of the formation of ZIF-67 nanoparticles within HMCSs via a solid-state reaction and subsequent selenization under a reducing atmosphere.

Firstly, HMCSs were synthesized as a conductive template according to previously reported methods [26]. The obtained 0.02 g of HMCSs was mixed with 0.2 mmol of Co(NO_3_)_2_·6H_2_O (97%, SAMCHUN) with the use of ethanol solvents. After drying, the sample was heat-treated at 60 °C under vacuum for 12 h with a ramping rate of 2 °C min^−1^. To form the ZIF-67 nanoparticles within HMCSs by means of the solid-state reaction, the cobalt-nitrate-loaded HMCSs were mixed with 2-MIM powder and heat-treated at 180 °C under vacuum for 1 h at a ramping rate of 10 °C min^−1^. Subsequently, the synthesized ZIF-67@HMCS composite was washed by filtration with ethanol several times to remove the unreacted 2-MIM. Finally, CoSe_2_@NC/HMCS composite was obtained via the selenization process of ZIF-67@HMCSs at 350 °C under a H_2_/Ar atmosphere for 6 h. For comparison, the composite of CoSe_2_ and HMCSs (denoted as CoSe_2_/HMCS) was prepared by means of a process similar to that for CoSe_2_@NC/HMCS composite except for 2-MIM addition. The experimental procedures for the characterization of the synthesized samples are described in detail in the Electronic Supplementary Material.

## Results and Discussion

### Synthesis and Characterization of CoSe_2_@NC/HMCS and CoSe_2_/HMCS Composites

**Scheme 1:**
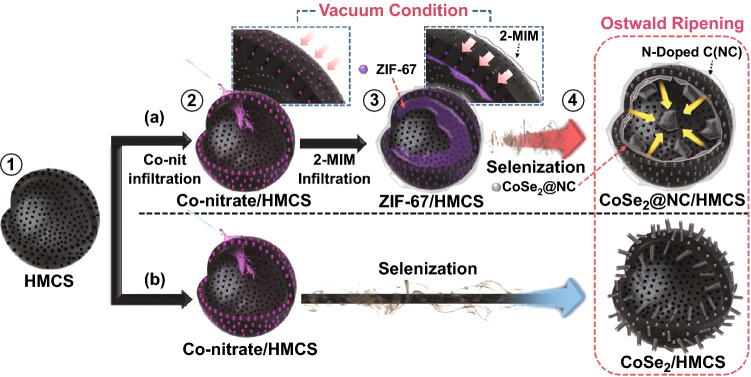
Schematic illustration for the preparation of CoSe_2_@NC/HMCS and CoSe_2_/HMCS composites

The procedure for synthesizing the uniquely structured CoSe_2_@NC/HMCS and CoSe_2_/HMCS composites is schematically depicted in Scheme 1. Firstly, HMCSs with a central void and mesoporous shell are prepared as a nanoreactor for confining CoSe_2_ nanocrystals (Scheme 1-①). Next, we use a vacuum-assisted infiltration method for the complete loading of MOFs into the HMCS templates. In particular, the powder obtained from drying the alcoholic solution containing Co(NO_3_)_2_·6H_2_O and HMCSs is heat-treated at a temperature over the melting point of Co(NO_3_)_2_·6H_2_O (55 °C) under vacuum (Scheme 1a-②). During this process, the melted Co-nitrate can easily infiltrate the well-developed mesopores of HMCS with vacuum assistance. Subsequently, a mixture of the Co-nitrate-infiltrated HMCSs and 2-MIM powder is further heated under vacuum (Scheme 1a-③) at a temperature of 180 °C, which is higher than the melting point of 2-MIM (145 °C). In this step, the thermal treatment under vacuum facilitates both the infiltration of 2-MIM into the HMCS and the simultaneous transformation of the mixture of the Co-nitrate and 2-MIM into stable ZIF-67 particles without requiring any solvent. During the subsequent selenization of ZIF-67/HMCS composite under H_2_/Ar conditions, the Co ions contained in the ZIF-67 can transform into CoSe_2_ nanoparticles, which are straightforwardly enclosed by the N-doped carbon matrix formed from the carbonization of 2-MIM, finally resulting in the formation of CoSe_2_@NC confined in HMCSs (Scheme 1a-④). For comparison purposes, we also synthesized CoSe_2_/HMCS composite through the direct selenization of Co-nitrate/HMCSs, as illustrated in Scheme 1b. We note that due to the Ostwald ripening phenomenon, most CoSe_2_ crystals protrude from the inner region of HMCSs to the outer surface, and they exhibit the shape of irregular rods or spheres.

The morphology and crystal structure changes of the samples obtained at each step were studied by means of various analytical tools. We note from Fig. S1a, b that the initially obtained monodisperse HMCSs exhibit not only numerous tiny pores on their 52-nm-thick shells but also a central void with a diameter of 330 nm; these plentiful pores and void can act as sites to effectively store the precursors. Via the vacuum-assisted method, the mesopores can be filled with Co-nitrate and 2-MIM precursors, a part of which is also deposited on the inner wall of the HMCS shell. Indeed, as confirmed from the scanning electron microscopy (SEM) and transmission electron microscopy (TEM) images (Figs. S1c–f and S2), the morphologies of the Co-nitrate/HMCS and ZIF-67/HMCS composites are very similar to that of the HMCS, and no aggregation is observed. Even after precursor loading, the central void of most HMCS can still clearly be discerned from the distinct contrast between the center and the edges, but with the HMCS shell becoming thicker (Figs. S1d, f, and S2a, b). The elemental mapping images in Fig. S2c depict the uniform distribution of C, N, and Co elements in the edge region of the shell, which indicates the formation of ZIF-67 on the inner surface of the HMCS shell. Furthermore, no peaks corresponding to ZIF-67 are observed in the X-ray diffraction (XRD) pattern of ZIF-67/HMCS, whereas the sample obtained from the solid-state reaction between only Co-nitrate and 2-MIM in the same weight ratio clearly shows the corresponding peaks (Fig. S3). These results prove that the ZIF-67 nanoparticles can be suitably confined in the HMCS templates via the vacuum-assisted method.

In order to demonstrate the difficulty in confining the MOF nanoparticles within the carbon templates with liquid-phase process, we newly prepared ZIF-67/HMCS composite via a liquid-phase process, and as shown in Fig. S4a, b, most of ZIF-67 nanoparticles were individually observed outside the HMCS template, which is quite different from the image of ZIF-67/HMCS composite prepared via a solid-state process. In light of these results, we believe that this solid-state process is an effective method to confine MOFs within the carbon templates. Also, we note that when the vacuum system is not used, some precursors cannot penetrate into inside and remain on the surface of HMCS as shown in Figs. S5 and S6 (SEM and TEM images). In particular, the sequential loading of Co-nitrate (Fig. S5a, b) and 2-MIM without applying vacuum leads to the formation of small-sized ZIF-67 nanoparticles on the outside of the HMCS (Figs. S5c, d, enlarged image, and S6a, b). The elemental mapping images in Fig. S6c depict the non-uniform distribution of C, N, and Co elements in the edge region of the shell, which revealed that the Co-nitrate and 2-MIM precursors cannot penetrate into HMCS under non-vacuum state.

**Fig. 1:**
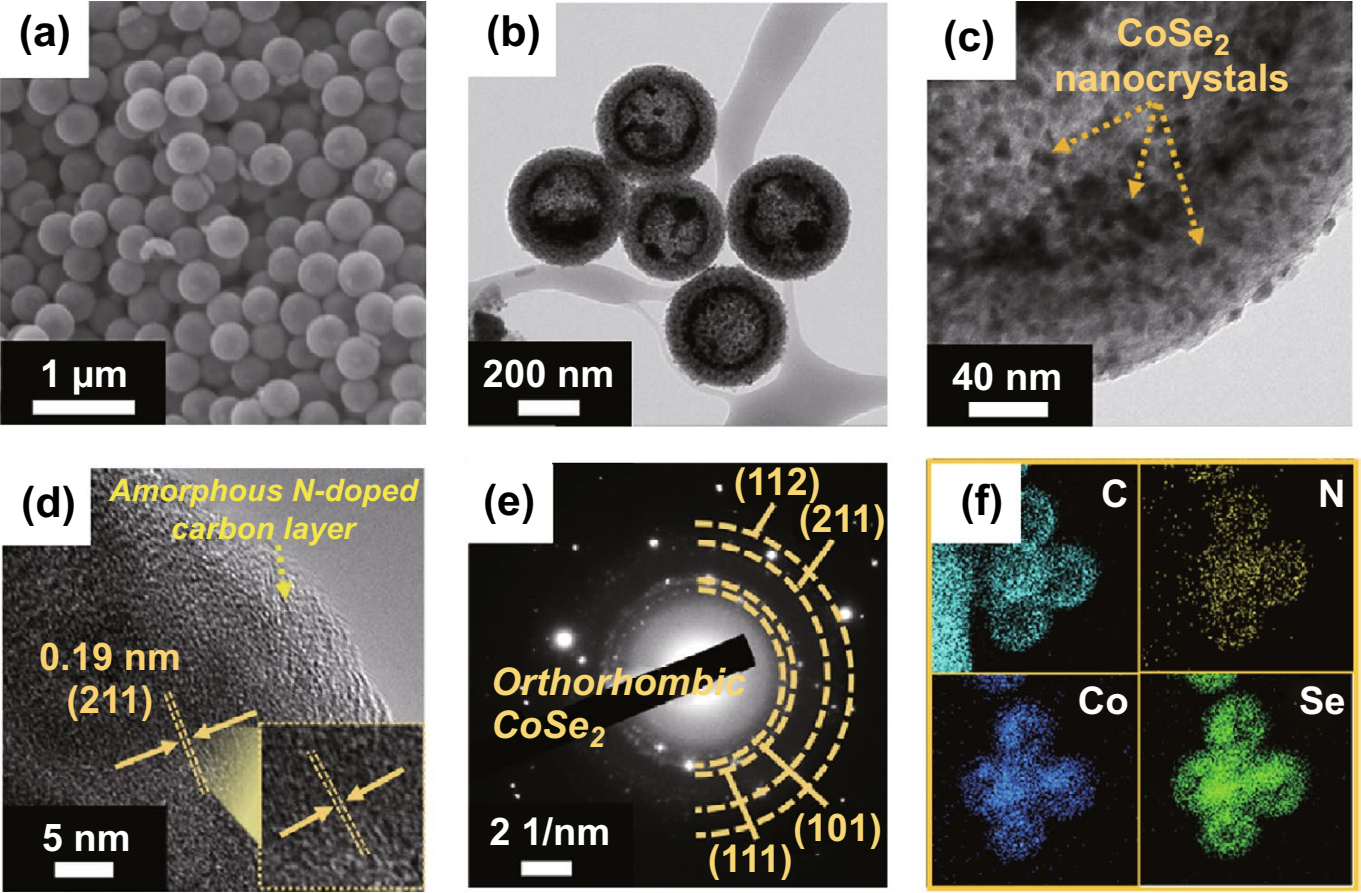
Morphologies, SAED pattern, and elemental mapping images of CoSe_2_@NC/HMCS composite formed with 2-methylimidazole: **a** SEM image, **b**, **c** TEM images, **d** HR-TEM image, **e** SAED pattern, and **f** elemental mapping images

After the selenization of ZIF-67/HMCS composite, the overall morphology does not differ significantly from that prior to the reaction, as shown in Fig. 1a. From the TEM images (Fig. 1b, c), we clearly observe the uniform distribution of ultrafine CoSe_2_ nanocrystals all over the HMCS, and the numerous mesopores in the HMCS shell are filled with nanocrystals with a diameter of ~ 5 nm. Moreover, large crystals in the central voids of several HMCSs are formed from the selenization of ZIF-67 deposited on the inner surface of the HMCS shell. During selenization, the initially formed small particles in the voids gradually grow via Ostwald ripening as the reaction progresses, thus resulting in the formation of fine CoSe_2_ crystals [27–29]. High-resolution TEM (HR-TEM, Fig. 1d) analysis shows the tiny nanocrystals enclosed by the amorphous carbon layer derived from the organic linkers of ZIF-67 along with lattice fringes separated by 0.19 nm corresponding to the (211) crystal planes, of orthorhombic CoSe_2_. Furthermore, the selected-area electron diffraction (SAED) and XRD patterns closely match with the orthorhombic CoSe_2_ crystal structure (Figs. 1e and S7). The elemental mapping images in Fig. 1f indicate the homogeneous distribution of C, N, Co, and Se elements. The presence of N evidences the formation of N-doped carbon from the organic linkers contained in ZIF-67 after selenization.

**Fig. 2:**
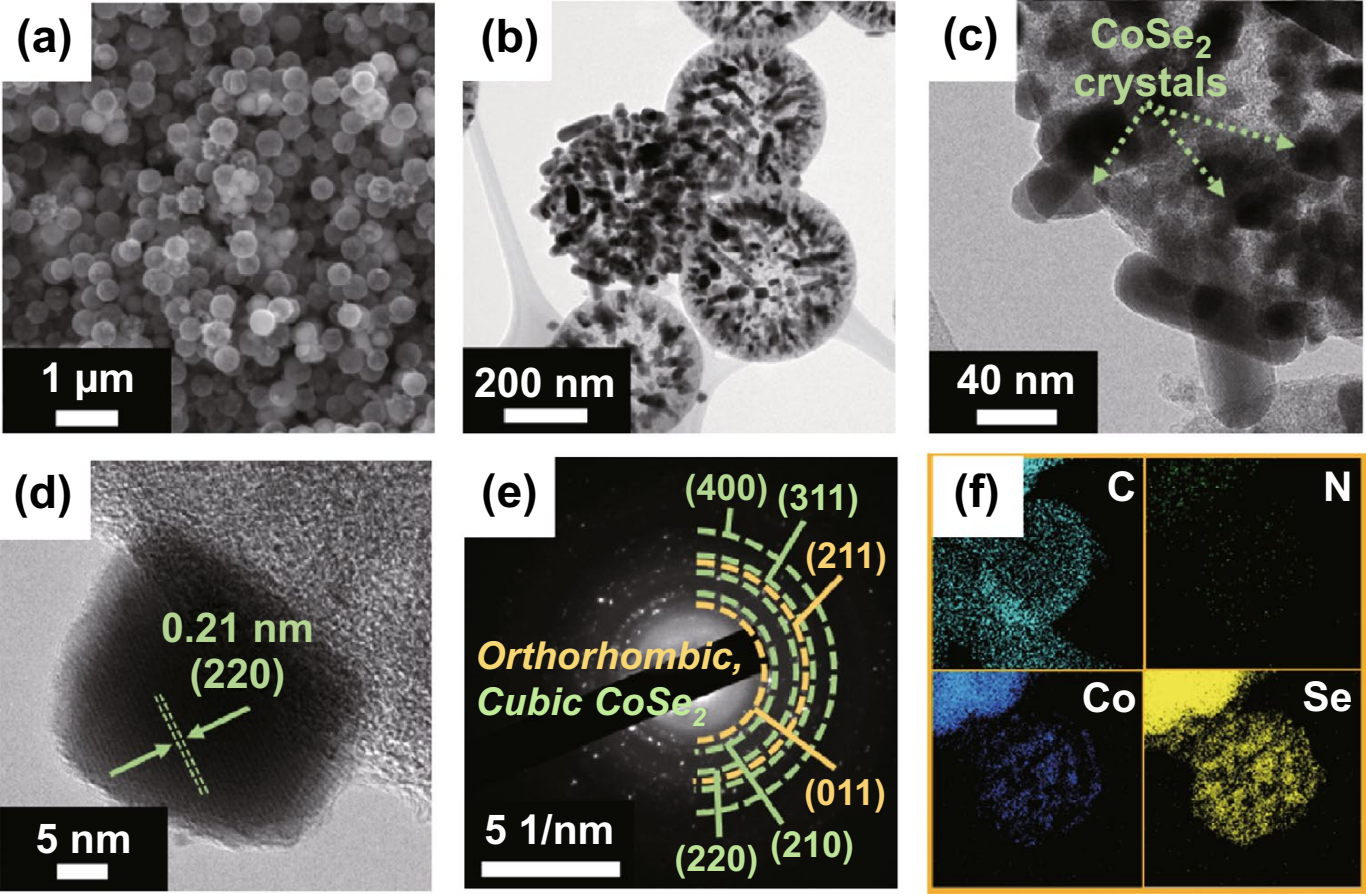
Morphologies, SAED pattern, and elemental mapping images of CoSe_2_/HMCS composite formed without 2-methylimidazole: **a** SEM image, **b**, **c** TEM images, **d** HR-TEM image, **e** SAED pattern, and **f** elemental mapping images

In contrast to the morphology of CoSe_2_@NC/HMCS, irregular-shaped nanocrystals are observed on the surface of the carbon nanospheres in the SEM image of the CoSe_2_/HMCS composite (Fig. 2a). Further TEM investigations (Fig. 2b, c) show the presence of overgrown nanoparticles and nanorods on the outside shell of the HMCS, which are larger than the nanocrystals of the CoSe_2_@NC/HMCS composite; these crystals and rods may be formed due to Ostwald ripening. Moreover, unlike the CoSe_2_@NC/HMCS composite, no carbon layer is observed on the surface of the protruded nanocrystals (Fig. 2d). These morphological and structural differences between CoSe_2_@NC/HMCS and CoSe_2_/HMCS composites prove that ZIF-67 formation can significantly suppress the overgrowth of CoSe_2_ nanocrystals during selenization. The SAED and XRD patterns reveal that the nanocrystals in the CoSe_2_/HMCS composite are composed of dominant cubic CoSe_2_ and minor orthorhombic CoSe_2_ crystalline phases (Figs. 2e and S7). From the elemental mapping images (Fig. 2f), there is no signal corresponding to N because of the absence of MOFs.

**Fig. 3:**
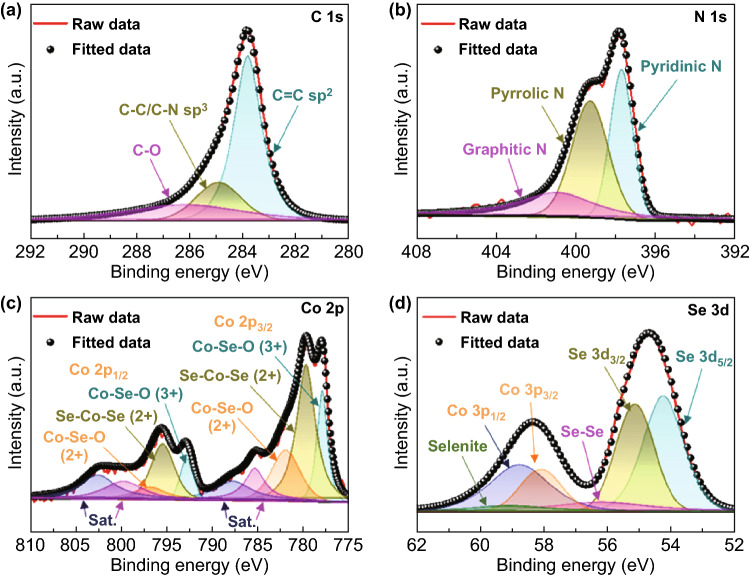
XPS spectra of CoSe_2_@NC/HMCS composite: **a** C 1s, **b** N 1s, **c** Co 2p, and **d** Se 3d

To study the chemical state of CoSe_2_@NC/HMCS composite, we performed the X-ray photoelectron spectroscopy (XPS) measurements (Figs. 3 and S8). From the XPS survey spectrum of the composite, the presence of C, N, O, Co, and Se elements is clearly confirmed, which agrees with the elemental mapping data (Fig. S8). The C 1 s spectrum shows deconvoluted peaks identified at 283.9, 284.9, and 286.2 eV, which are assigned to C = C (*sp*^2^), C–C/C–N (*sp*^3^), and C–O bonds, respectively (Fig. 3a) [14, 30]. For the N 1 s spectrum, three peaks corresponding to pyridinic, pyrrolic, and graphitic N can be confirmed at 397.7, 399.3, and 400.9 eV, respectively (Fig. 3b) [6, 31]. Based on the XPS quantitative results, the N content in the composite was calculated to be 12.9 at %. Meanwhile, the high-resolution Co 2p spectrum is deconvoluted into many peaks corresponding to Se–Co–Se bonds, cobalt selenites, and satellites (indicated as Sat.) (Fig. 3c) [32, 33]. In a previous report, the presence of peaks corresponding to metal selenites was attributed to the formation of Co–Se–O bonds by the partial surface oxidation of metal selenides [34]. In our case, the high-resolution XPS spectrum of Se 3d can be fitted well with the peaks corresponding to Se 3d_5/2_, Se 3d_3/2_, Se–Se (metalloid Se), Co 3p_3/2_, Co 3p_1/2_, and selenite (Fig. 3d) [14, 35, 36]. The presence of the Se–Se peak indicates that trace metalloid Se remains in the composite. Moreover, the partial surface oxidation of metalloid Se is responsible for the presence of the cobalt selenite peak.

From the thermalgravimetric analysis (TGA) analysis curves of CoSe_2_@NC/HMCS and CoSe_2_/HMCS composites (Fig. S9a), we observe multiple-step weight losses in the range of 300–600 °C; these can be mainly attributed to the combustion of carbonaceous materials and the oxidation of CoSe_2_ nanocrystals. Based on the TG results, the calculated carbon amounts in CoSe_2_@NC/HMCS and CoSe_2_/HMCS composites are 64% and 43%, respectively. From the Raman spectroscopy result (Fig. S9b), the *I*_D_/*I*_G_ value of CoSe_2_@NC/HMCS can be determined as 1.05, which indicates defects in the carbon structure, possibly due to N doping and incomplete graphitization. Next, using the Brunauer–Emmett–Teller (BET) analysis (Fig. S9c, d), we calculated the specific surface areas of HMCS, CoSe_2_@NC/HMCS, and CoSe_2_/HMCS composites to be 900.1, 77.2, and 46.6 m^2^ g^−1^, respectively. When compared with that of the CoSe_2_/HMCS composite, the higher surface area of the CoSe_2_@NC/HMCS composite can be attributed to the presence of porous carbon derived from MOF carbonization. The relatively low surface areas and pore volumes of the composite indicate that CoSe_2_ nanocrystals are well embedded in the HMCSs.

### K-Ion Storage Mechanism and KIB Performances

**Fig. 4:**
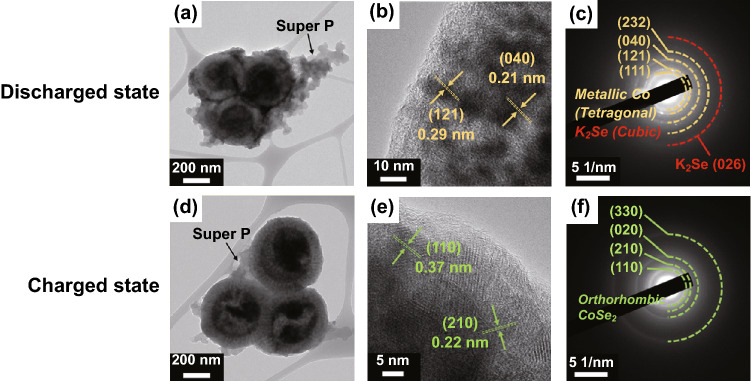
TEM images and SAED patterns of CoSe_2_@NC/HMCS composite at the fully **a-c** discharged and **d-f** charged states: **a**, **b**, **d**, **e** TEM images and **c**, **f** SAED patterns

**Fig. 5:**
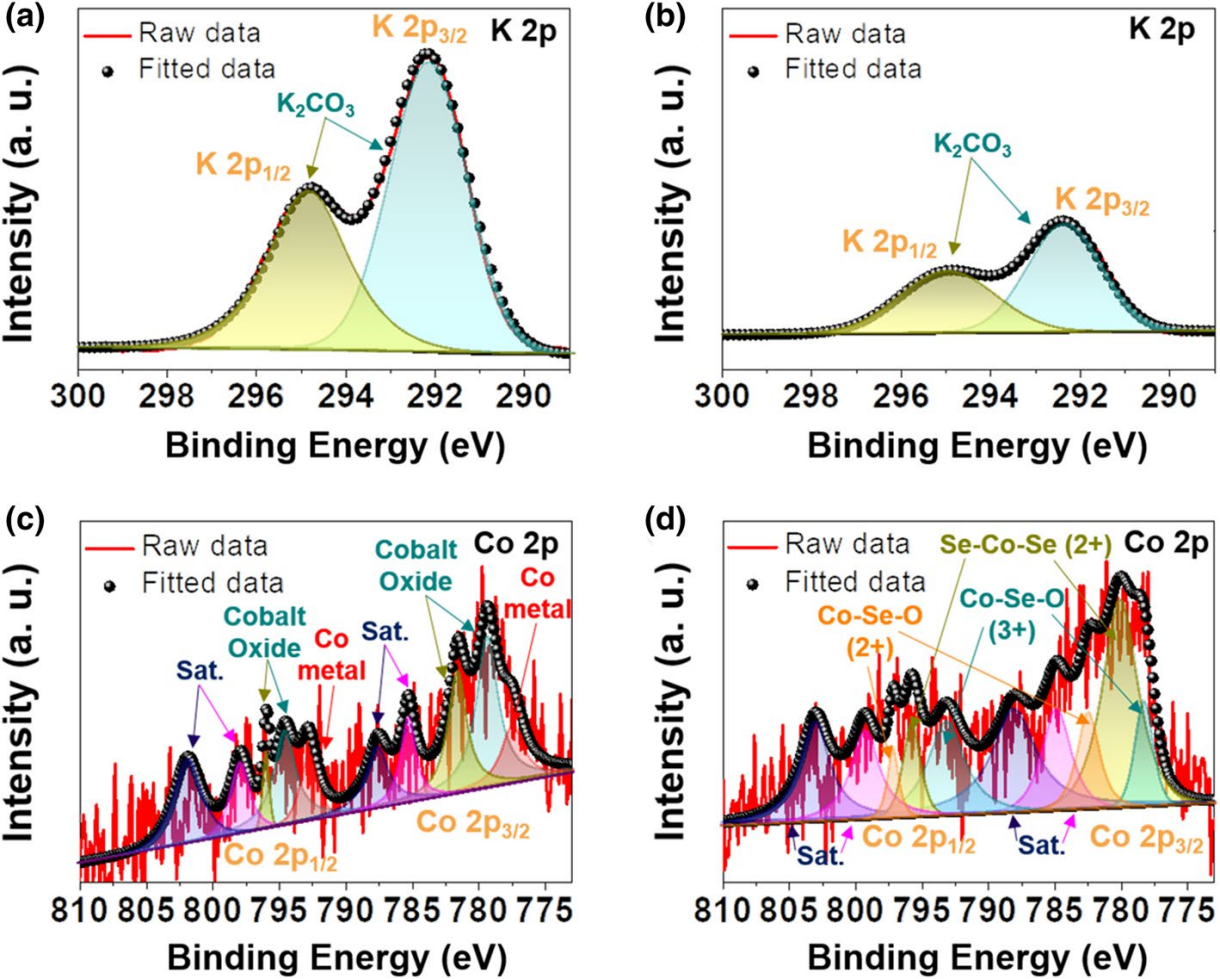
XPS spectra of CoSe_2_@NC/HMCS composite: **a**, **b** K 2p, **c**, **d** Co 2p measured after **a**, **c** the initial fully discharged state and **b**, **d** the initial fully charged state

Considering that very few studies have focused on CoSe_2_ embedded in a porous carbon matrix for application as KIB anodes, we investigated the potassium storage mechanism of CoSe_2_@NC/HMCS based on the ex situ analysis of the initial fully discharged/charged electrodes (Figs. 4 and 5). The TEM images of the fully discharged and charged composite are not as clear as those of the uncycled composite, which could be due to the formation of solid electrolyte interphase (SEI) layers (Fig. 4a, d). From the HR-TEM image and SAED pattern of the electrode in the fully discharged state (Fig. 4b and c, respectively), we clearly observe the formation of tetragonal-phase metallic Co nanoparticles and cubic-phase K_2_Se. These results indicate that cobalt selenide nanocrystals transform into the corresponding metallic Co nanoparticles, and K_2_Se is simultaneously formed as a by-product of the chemical reaction between K and Se ions [37]. After full charging, the re-formation of orthorhombic-phase CoSe_2_ in the composite can be confirmed from the corresponding HR-TEM image and SAED pattern (Fig. 4e, f, respectively); these results indicate that the metallic Co nanoparticles revert back to CoSe_2_ after the charging process. The ex situ XPS results also support the hypothesized electrochemical storage mechanism of the CoSe_2_@NC/HMCS electrode [38]. In high-resolution K 2p spectrum, the K_2_CO_3_ peak intensity in the fully discharged electrode is higher than that in the fully charged electrode (Fig. 5a, b), which could be due to the partial decomposition of the SEI layer composed of K_2_CO_3_ and other by-products [39, 40]. Moreover, from the Co 2p spectrum of the fully discharged electrode, we can clearly identify the deconvoluted peak assigned to metallic Co, which disappears after subsequent charging (Fig. 5c, d). Instead, the Se–Co–Se bond peaks are observed again, which is also supported by the TEM results. The peaks corresponding to cobalt oxides can be ascribed to the partial surface oxidation of cobalt during the measurement.

**Fig. 6:**
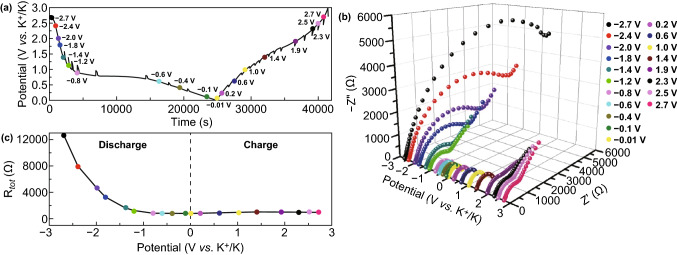
In situ EIS of CoSe_2_@NC/HMCS composite: **a** preselected potentials and time curve at a current density of 0.1 A g^−1^, **b** in situ Nyquist plots at predetermined potentials, and **c** in situ EIS graph (*R*_*tot*_ vs. potential) measured at preselected potential during the initial cycle

Figure 6 shows the in situ electrochemical impendence spectroscopy (EIS) analysis results of the CoSe_2_@NC/HMCS electrode at various voltage levels during the initial cycling; the EIS results further support the electrochemical mechanism revealed by the cyclic voltammetry (CV) graphs. The Nyquist plots are fitted by the Randles-type equivalent circuit model (Fig. S10) and the plots of the synthesized sample are related to the electrolyte resistance (*R*_s_), interfacial resistances (*R*_sei_), charge transfer resistance (*R*_ct_), and ion diffusivity, which is drawn as a semicircle and sloping line on the graph. The points in Fig. 6a indicate the preselected potentials at which the in situ EIS results were obtained. The Nyquist plots of CoSe_2_@NC/HMCS at the preselected potentials are depicted in Fig. 6b and the *R*_tot_ change (related to the sum of *R*_s_, *R*_sei_, and *R*_ct_) is indicated in Fig. 6c. In the initial discharging process, the *R*_tot_ value at the predetermined potentials gradually decreases until − 0.6 V. In this potential range, the CoSe_2_@NC/HMCS composite transforms into ultrafine metallic Co and K_2_Se, and the potassium bis(fluorosulfonyl) imide (KFSI) in the electrolyte forms a stable SEI layer on the electrode, which can reduce the electrode resistance [41, 42]. However, the *R*_tot_ value slightly increases during the subsequent charging process until 1.9 V because of the structural stress caused by the phase transformation of metallic Co to CoSe_2_. Subsequently, *R*_tot_ continues to decrease until a preselected final potential, which may be associated with complete depotassiation and the decomposition of the reversible SEI layer [43–45]. These results are in good agreement with the above-mentioned ex situ TEM and XPS data (Figs. 4 and 5).

**Fig. 7:**
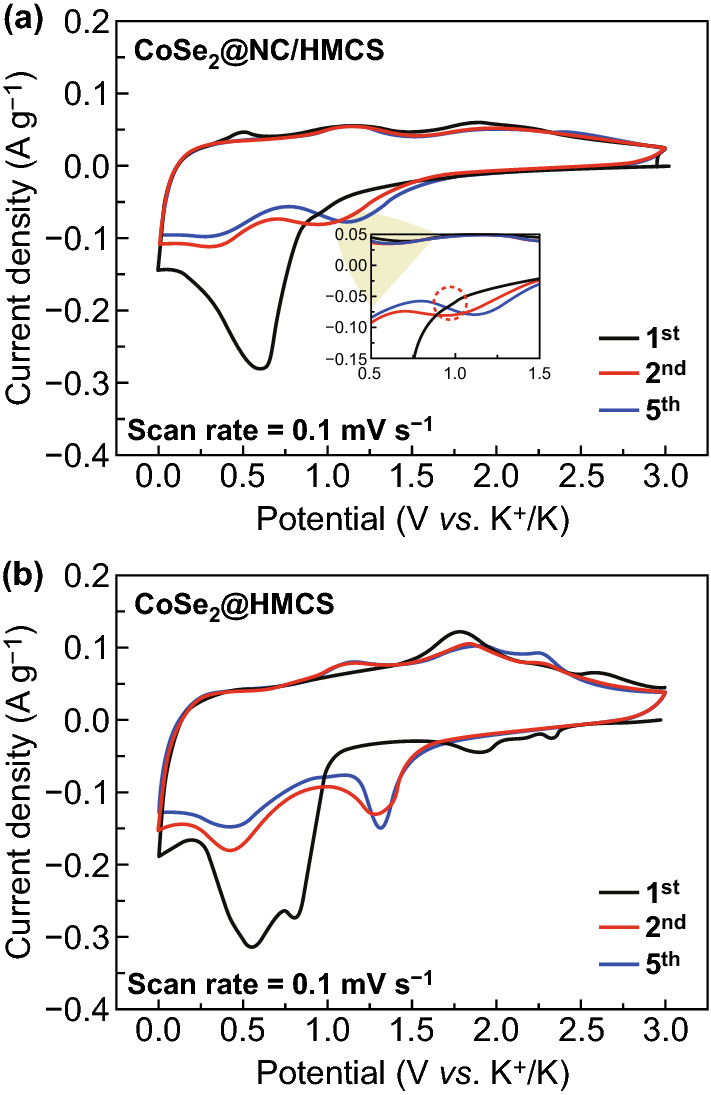
CV curves of **a** CoSe_2_@NC/HMCS and **b** CoSe_2_/HMCS composites obtained at a scan rate of 0.1 mV s^−1^

We next applied the CV technique over the potential range of 0.001–3.0 V (vs. K^+^/K) to confirm the electrochemical behaviors of CoSe_2_@NC/HMCS and CoSe_2_/HMCS electrodes when used as an anode in KIBs. In Fig. 7, the CV graphs of these two anodes for the first, second, and fifth cycles exhibit similar plots that indicate their similar redox reactions. During the initial cathodic sweep, the graph of CoSe_2_@NC/HMCS shows distinct peaks at ~ 0.95 and 0.60 V, corresponding to the reduction reaction between K^+^ and CoSe_2_ crystals and the formation of SEI layers, respectively (Fig. 7a) [10, 46]. The peak at ~ 0.01 V is correlated to the storage of K^+^ in the carbon shell [47]. These data are consistent with the results of the ex situ analysis. From the second cycle onward, the CV graph shifts forward due to the formation of tiny CoSe_2_ particles after the initial cycle. This causes the existing peaks to disappear and two new peaks at ~ 0.94 and 0.3 V to appear. The peak at ~ 0.94 V is related to the insertion of K^+^ into CoSe_2_ crystals and that at ~ 0.3 V is correlated with the conversion reaction with further K^+^. In the initial anodic sweep, the three peaks at ~ 1.89, 1.07, and 0.5 V appear on the CV graph. The peaks at ~ 1.89 and 1.07 V are related to the transformation of metallic Co nanoparticles and K_2_Se into CoSe_2_ nanocrystals [10, 32]. The peak at ~ 0.5 V corresponds to the release of K^+^ ions that enter the carbon shell during the discharging process [48]. The CV graphs of the second and fifth cycles exhibit a strong overlap, which indicates good electrochemical stability of the CoSe_2_@NC/HMCS composite. Additionally, the CoSe_2_/HMCS composite exhibits more distinct peaks in the CV graphs (Fig. 7b) during the charge–discharge process because of the lesser amount of carbonaceous materials relative to that of CoSe_2_@NC/HMCS, which can be related to the large crystal growth (Fig. 2d).

**Fig. 8:**
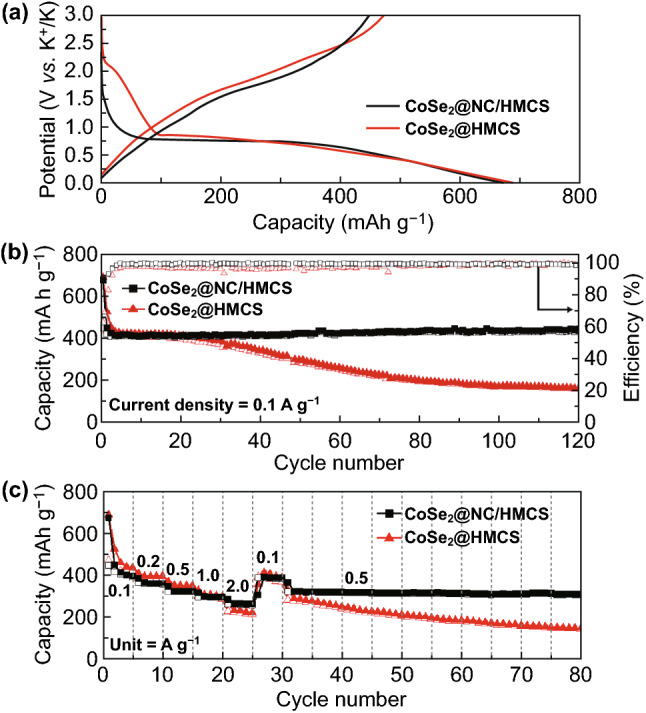
Electrochemical properties of CoSe_2_@NC/HMCS and CoSe_2_/HMCS composites: **a** initial galvanostatic charge–discharge curves, **b** cycle performances at a current density of 0.1 A g^−1^, and **c** rate performances at various current densities

The electrochemical performances of the CoSe_2_@NC/HMCS and CoSe_2_/HMCS composites are shown in Fig. 8. In the initial charge–discharge profiles of both electrodes at a current density of 0.1 A g^−1^ (Fig. 8a), the potential level of the distinct long plateaus is in close agreement with the peak locations in the CV graphs. The initial discharge capacities of CoSe_2_@NC/HMCS and CoSe_2_/HMCS composites are 675 and 689 mAh g^−1^, the initial charging capacities are 448 and 472 mAh g^−1^, and the initial coulombic efficiencies (CEs) are 66% and 68%, respectively. The low values of initial capacity and CE are mainly related to the formation of the SEI layers by electrolyte decomposition, which is proportional to the electrode surface area [49, 50]. Figure 8b shows the cycle performances of CoSe_2_@NC/HMCS and CoSe_2_/HMCS electrodes at a current density 0.1 A g^−1^ for 120 cycles. Over the entire cycle, CoSe_2_@NC/HMCS shows a high reversible discharge capacity of 442 mAh g^−1^ for 120 cycles with good cycling stability (almost 99% CEs all over the cycling). On the other hand, in the case of the CoSe_2_/HMCS electrode, a rapid cycle decay is observed after 30 cycles, and eventually, the reversible discharge capacity reaches 160 mAh g^−1^ after 120 cycles. This result indicates that the active material of the electrode could be pulverized due to the large volume expansion during the repeated charging and discharging process. When compared with the CoSe_2_@NC/HMCS electrode, there is more aggregation between particles, and a thicker SEI layer is formed in CoSe_2_/HMCS electrode after 100 cycles (Fig. S11a-d). Furthermore, to confirm the electrochemical behavior and K-ion storage contribution of the HMCS in composite, we analyzed the electrochemical properties under the same conditions (Fig. S12a-c). As confirmed in Fig. S12a, HMCS showed the charge–discharge curves of a typical carbonaceous material; there were no apparent plateaus. They also delivered a specific discharge capacity of 208 mAh g^−1^ after 50 cycles at a current density of 0.1 A g^−1^ (Fig. S12b). Despite their low specific capacities, the synergistic effect between CoSe_2_ and the conductive HMCS template with mesopores enhanced the capacity of the composite.

Meanwhile, CoSe_2_@NC/HMCS electrodes were further tested at the ranges of 0.001–2.0 V and 0.001–2.5 V to confirm the effect of potential window range on the electrochemical performance, (Fig. S13a-c). The result exhibited larger initial irreversible capacity and lower reversible capacities than those evaluated at the range of 0.001–3.0 V (Fig. 8), which indicates that the redox reaction may have not occurred sufficiently in the narrow potential range. In light of these results, we make a conclusion that the potential range of 0.001–3.0 V is optimal test condition for CoSe_2_@NC/HMCS electrodes.

Figure 8c illustrates the rate performance of the two composites in the range of 0.1 to 2.0 A g^−1^. Although the CoSe_2_/HMCS composite delivers stable capacities in the low-current–density regime, the capacity begins to gradually decrease from 1.0 A g^−1^. In addition, when the current density again reverts to 0.5 A g^−1^, the capacity barely recovers. In contrast, the CoSe_2_@NC/HMCS composite delivers stable reversible capacities for all current densities, thereby indicating excellent rate capability. The reversible discharge capacities of CoSe_2_@NC/HMCS composite are 394, 363, 324, 298, and 263 mAh g^−1^ at current densities of 0.1, 0.2, 0.5, 1.0, and 2.0 A g^−1^, respectively. Moreover, after the current density reverts to low values of 0.1 and 0.5 A g^−1^, the reversible capacity values are almost completely restored, and good cycling stability is observed for up to 80 cycles. Also, in Fig. S14, the charge–discharge curves of CoSe_2_@NC/HMCS have similar shapes without significant change at even high current density, which verifies excellent rate performance of CoSe_2_@NC/HMCS electrode. On the other hand, CoSe_2_@NC/HMCS composite prepared from the precursors obtained under non-vacuum state showed poor cycling and rate performances (Fig. S15). This could be because CoSe_2_@NC particles in the composite are not confined within HMCS and are directly exposed to the electrolyte. The aggregation and overgrowth of active material can easily induce structure collapse during repeated cycling process.

To further investigate the influences amount of Co-nitrate in the composite, we added the SEM images and electrochemical performance data of two comparison samples obtained by only adjusting the amount of the Co-nitrate (Figs. S16 and S17). When synthesized by adding only 1/3 times the original amount of Co-nitrate (denoted as CoSe_2_@NC/HMCS-1/3), there was little difference in the morphology from the CoSe_2_@NC/HMCS (Fig. S16a). On the other hand, excessive addition of Co-nitrate beyond the absorbing capacity of HMCS template (3 times the original amount, CoSe_2_@NC/HMCS-3) caused aggregation of CoSe_2_@NC particles outside of HMCS (Fig. S16b). Additionally, two samples were galvanostatically evaluated as anodes for KIB to confirm their electrochemical properties (Fig. S17). In the cycling performance data, CoSe_2_@NC/HMCS-1/3 exhibited lower specific capacity than CoSe_2_@NC/HMCS, whereas CoSe_2_@NC/HMCS-3 showed higher specific capacity over the entire cycles (Fig. S17a). Meanwhile, despite the highest capacity value of CoSe_2_@NC/HMCS-3, its rate capability was the worst. At a high current density of 2.0 A g^−1^, the capacity of CoSe_2_@NC/HMCS-3 decreased more rapidly compared to other samples, and even after the current density returned to 0.5 A g^−1^, the capacity of CoSe_2_/HMCS continued to decrease (Fig. S17b). This could be due to the CoSe_2_@NC aggregates in the composite that are not confined within HMCS. On the contrary, the other samples showed stable capacity retention throughout all current densities.

These outstanding electrochemical performances of the CoSe_2_@NC/HMCS composite as KIB anodes can be attributed to its several distinct characteristics. First, the HMCS template and N-doped carbon matrix provide both structural robustness and high electronic conductivity. Second, the unique porous structure consisting of the central void and mesopores can improve the ion diffusion rate and accessibility. Finally, the formation of ultrafine CoSe_2_ nanocrystals by the “dual confinement system” can effectively alleviate volume expansion during the cycling. In comparison with previously reported metal-selenide-based anodes for KIBs, our CoSe_2_@NC/HMCS composite exhibits superior electrochemical performances (Table S1), particularly in terms of the reversible capacity and cycling stability.

**Fig. 9:**
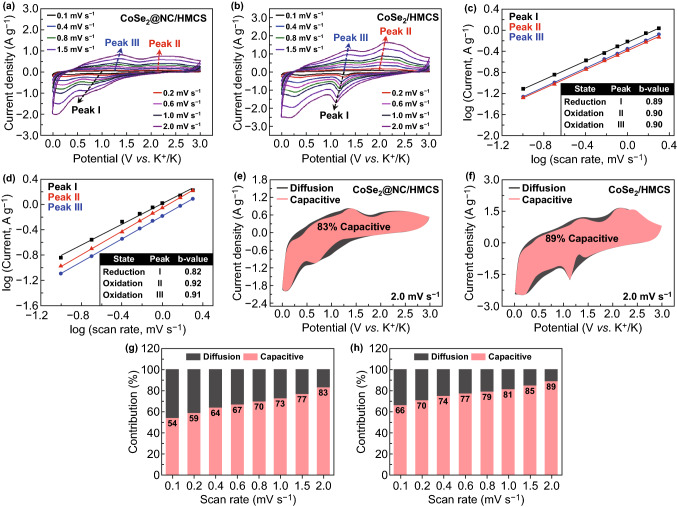
Kinetic analysis of **a**, **c**, **e**, **g** CoSe_2_@NC/HMCS and **b**, **d**, **f**, **h** CoSe_2_/HMCS electrodes for KIBs: **a**, **b** cyclic voltammograms at various sweep rates, **c**, **d** fitted log (peak current) vs. log (scan rate) for peaks corresponding to certain electrochemical reactions, **e**, **f** cyclic voltammograms showing capacitive contribution (colored area) at a scan rate of 2.0 mV s^−1^, and **g**, **h** capacity contribution at various scan rates

To examine the electrochemical kinetics, we performed CV measurements of the two electrodes at various scan rates ranging from 0.1 to 2.0 mV s^−1^ (Fig. 9). We note that as the scan speed increases, the cathodic and anodic peaks of the composites gradually deviate from the original peak position due to ohmic resistance (Fig. 9a, b) [9]. The relationship between the current (i) and scan rate (v) can be expressed as follows: i = aν^b^, where both a and b represent empirical values [51, 52]. From Fig. 9c, d, we note that the b values of both electrodes are closer to 1.0, indicating that the capacitive-controlled process is dominant during the charge–discharge process of the two composites [53]. The correlation graphs between the scan rate and capacitive contribution show that the capacitive contributions of CoSe_2_@NC/HMCS and CoSe_2_/HMCS composites gradually increase with the scan rate, and they reach 83% and 89%, respectively, at the scan rate of 2.0 mV s^−1^ (Fig. 9e-h). These high capacitive contributions indicate the quick charge/discharge properties of CoSe_2_@NC/HMCS and CoSe_2_/HMCS electrodes. However, despite their high capacitive contribution, the CoSe_2_/HMCS electrode exhibited a relatively poor rate performance. This is because the rate capability can be influenced not only by several factors related to capacitive behavior, but also by structural change. Therefore, the poor rate capability of CoSe_2_/HMCS electrode could be due to the structural collapse at high current densities.

Next, we conducted EIS measurements to confirm the resistance value changes of the electrodes for fresh, 1st, 60th, and 100th cycles (Fig. S18). In Fig. S18a, the CoSe_2_@NC/HMCS electrode exhibits a smaller Nyquist plot than the CoSe_2_/HMCS electrode in the fresh state, thus indicating that the CoSe_2_@NC/HMCS electrode has a lower charge transfer resistance. After the initial cycle, the *R*_ct_ values of the two electrodes drastically decrease, which can be ascribed to the formation of smaller CoSe_2_ nanocrystals during the cycling and electrode activation by the electrolyte [54, 55]. The *R*_ct_ value of CoSe_2_@NC/HMCS electrode gradually decreased as cycle proceeds, indicating the structural robustness of the electrode (Fig. S18b). In contrast, the *R*_ct_ value of the CoSe_2_/HMCS electrode at the 100th cycle increases over that at the 60th cycles (Fig. S18c), which could be due to structural collapse during the charge–discharge process. These results are in good agreement with the cycle data shown in Fig. 8b. The relationship between the phase angle (ω^−1/2^) and impedance (Z’) of the two electrodes at the 100th cycle is plotted in Fig. S18d. The cycled CoSe_2_@NC/HMCS electrode exhibits a smaller slope than that of the cycled CoSe_2_/HMCS electrode, thereby indicating a faster K^+^ ion diffusion rate after 100 cycles [56].

## Conclusions

In summary, we synthesized a MOF-derived ultrafine CoSe_2_ nanocrystal@NC matrix confined in hollow mesoporous carbon nanospheres for application as the anode material of high-performance KIBs. The application of thermal treatment under vacuum state leads to not only the easy infiltration of precursors but also the immediate formation of ZIF-67 nanoparticles within HMCSs. This novel approach can effectively suppress the overgrowth of CoSe_2_ nanocrystals during the subsequent selenization step, thereby resulting in the formation of ultrafine CoSe_2_ nanocrystals embedded in the N-doped carbon matrix and their homogeneous distribution within the HMCSs. These unique features ensure that the CoSe_2_@NC/HMCS composite can not only provide sufficient channels for electron and ion transfers but also alleviate the volume expansion of CoSe_2_ nanocrystals during electrochemical reactions. Accordingly, the CoSe_2_@NC/HMCS composite exhibited a high reversible capacity, long-term cycling stability, and excellent rate capabilities when used as an anode for KIBs. Our strategy for synthesizing uniquely structured metal selenide/carbon composite can be extended to other novel electrodes for high-performance energy storage applications.

## Electronic supplementary material

Below is the link to the electronic supplementary material.

Supplementary material 1 (PDF 2071 kb)
