# Supplementary material for: MOF-Derived CoSe2@N-Doped Carbon Matrix Confined in Hollow Mesoporous Carbon Nanospheres as High-Performance Anodes for Potassium-Ion Batteries
Source: Nanomicro Lett. 2020 Oct 27;13:9. doi: 10.1007/s40820-020-00539-6 (PMC8187686; doi:10.1007/s40820-020-00539-6)
Supplement: Supplementary file 1 — Supplementary material 1 (PDF 2071 kb) [file 40820_2020_539_MOESM1_ESM.pdf]

Supporting Information for

MOF-Derived $CoSe_2$@N-Doped Carbon Matrix Confined in Hollow Mesoporous Carbon Nanospheres as High-Performance Anodes for Potassium-Ion Batteries

Su Hyun Yang[1, +], Seung-Keun Park[2, +], Yun Chan Kang[1, *]

[1]Department of Materials Science and Engineering, Korea University, Anam-Dong, Seongbuk-Gu, Seoul 136-713, Republic of Korea

[2]Department of Chemical Engineering, Kongju National University, 1223-24 Cheonan-daero, Seobuk-gu, Cheonan, 31080, Republic of Korea

[+]Su Hyun Yang and Seung-Keun Park contributed equally to this work.

*Corresponding author. E-mail: yckang@korea.ac.kr (Yun Chan Kang)

S1 Supplementary Characterizations of Materials

The morphologies and full structures of the synthesized samples were investigated by means of scanning electron microscopy (SEM, VEGA3 SBH) and field-emission transmission electron microscopy (FE-TEM, JEM-2100 F). The *ex-situ* TEM analysis of $CoSe_2$@NC/HMCS composites in the fully discharged and charged states was conducted using the same equipment. The sample crystallographic features were confirmed with the use of powder X-ray diffraction (XRD, RIGAKU D/MAX-2500V) with Cu-Kα radiation (λ = 1.5418 Å) at Korea University (Seoul). X-ray photoelectron spectroscopy (XPS, Thermo Scientific K-Alpha) was used to measure the chemical content of the composites, and a Pyris 1 thermogravimetric (TG) analyzer (Perkin Elmer) was used to confirm the carbon content of the composite in the temperature range of 30–700 °C at a ramping rate of 10 $°C·min^{-1}$ in air. *Ex-situ* XPS analysis of the electrodes in the fully discharged and charged states was performed by using the ULVAC-PHI X-TOOL. The pore sizes and surface areas of the prepared materials were evaluated by using the Brunauer–Emmett–Teller (BET) method, with pure N_2 as the adsorbate gas. Raman spectroscopy (Jobin Yvon LabRam HR800, samples excited by a 632.8-nm He/Ne laser) was conducted to analyze the carbon structure in the composites.

S2 Supplementary Electrochemical Measurements

The electrochemical properties of the $CoSe_2$@NC/HMCS and $CoSe_2$/HMCS composites were examined with the use of a standard 2032-type coin cell. The potassium-ion battery (KIB) anodes were fabricated by mixing the active material, Super P, and sodium carboxymethylcellulose (weight ratio of 7:2:1, respectively) in DI water, and the mixture was then applied onto copper foil using a doctor blade. The coin cell consisted of potassium metal as the counter-electrode, porous polypropylene

as the separator, and 1 M potassium bis(fluorosulfonyl) imide (KFSI) dissolved in a mixture of ethylene carbonate/diethyl carbonate (EC/DEC, volumetric ratio of 1:1) as the electrolyte, with the cell being manufactured in a glove box. The galvanostatic charge/discharge characteristics and cyclic voltammetry (CV) determinations were conducted by using a battery analyzer (WonATech, WBCS-3000s cycler) over the potential range of 0.001–3.0 V at various current densities. The diameter and mass loading of the electrode were 1.4 cm and 1.2–2.0 mg cm^{-2}, respectively. Electrochemical impendence spectroscopy (EIS) measurements of the coin cell were conducted in the range of 0.01–100 kHz.

S3 Supplementary Figures and Table

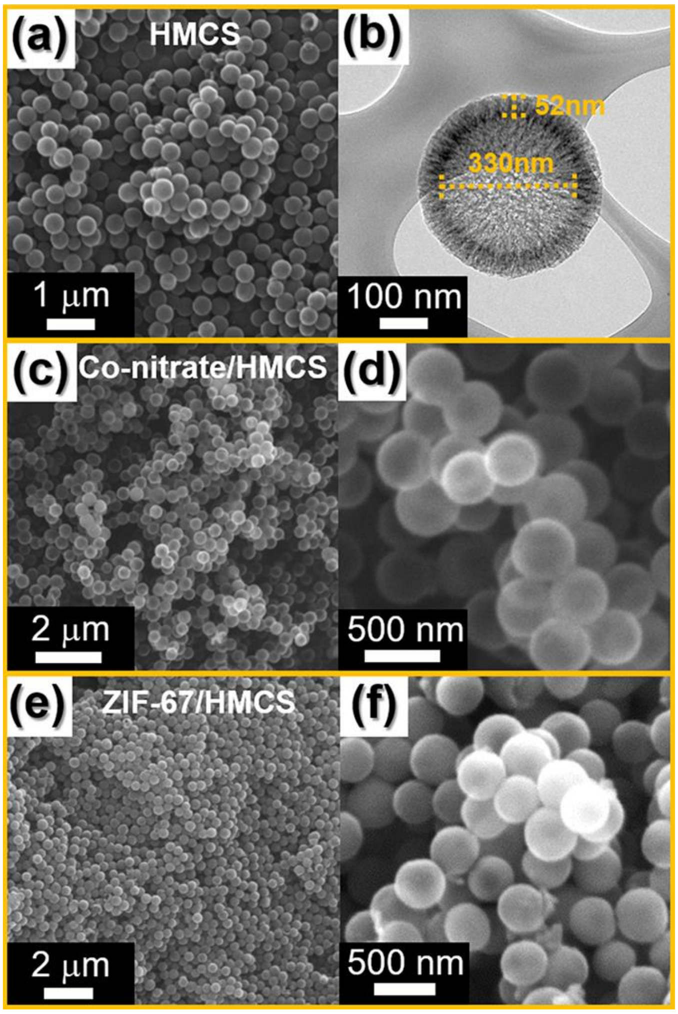

Fig. S1 Morphologies of HMCS, Co-nitrate/HMCS, and ZIF-67/HMCS prepared under vacuum state : **a, b** SEM image and TEM image of HMCS, **c, d** SEM images of Co-nitrate/HMCS, and **e, f** SEM images of ZIF 67/HMCS

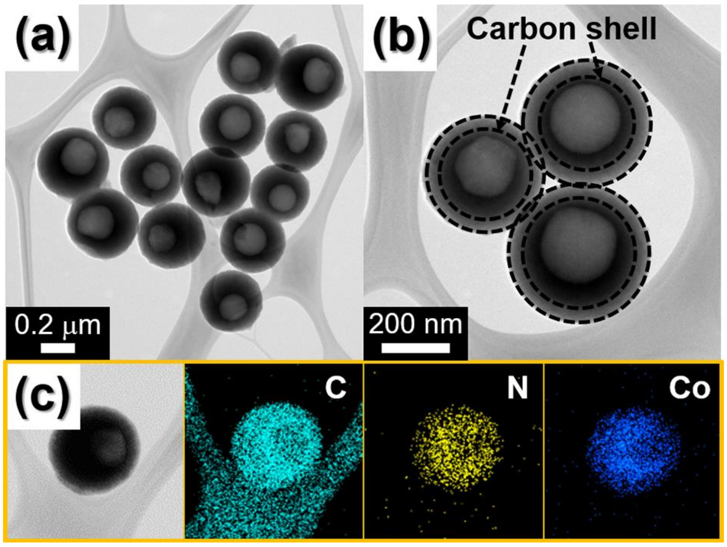

Fig. S2 Morphologies and elemental mapping images of ZIF-67/HMCS composite prepared under vacuum state: **a, b** TEM images and **c** elemental mapping images

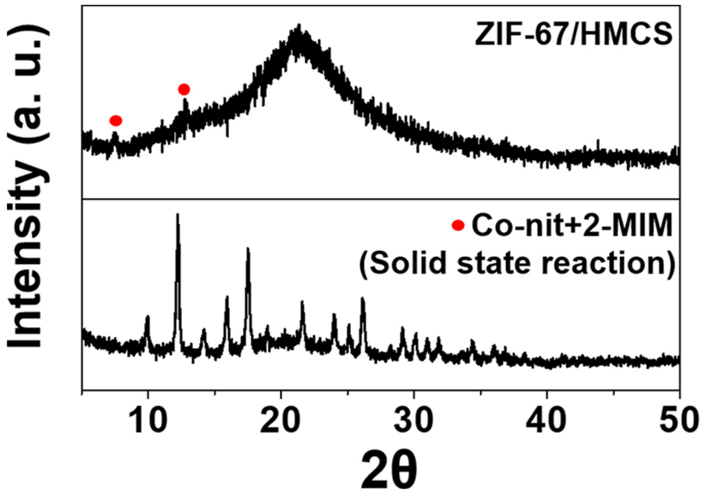

Fig. S3 XRD patterns of ZIF-67/HMCS composite and powders formed by solid-state reaction of cobalt salt and 2-methylimidazole at 180 °C

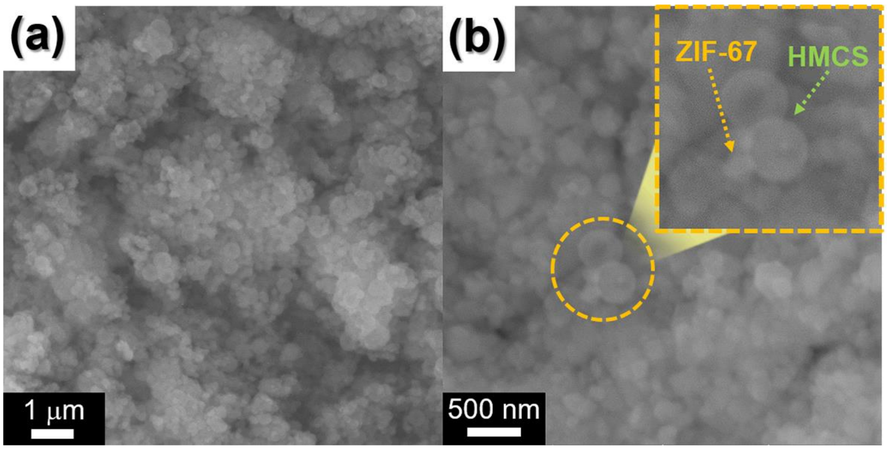

Fig. S4 Morphologies of ZIF-67/HMCS composite prepared by liquid-phase process: **a, b** SEM images

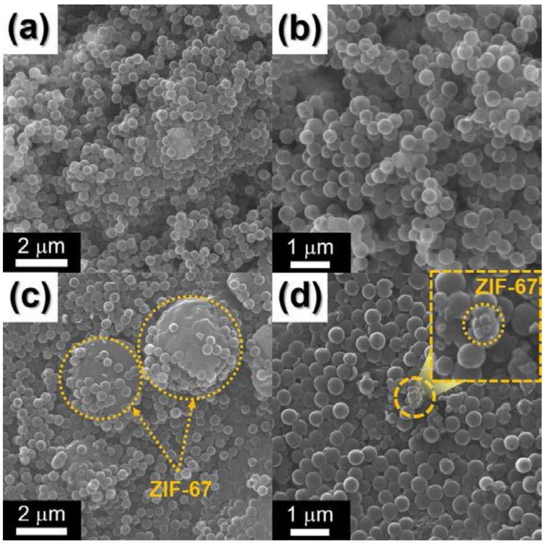

Fig. S5 Morphologies of Co-nitrate/HMCS and ZIF-67/HMCS composites synthesized under non-vacuum state: **a, b** SEM images of Co-nitrate/HMCS and **c, d** SEM images of ZIF-67/HMCS

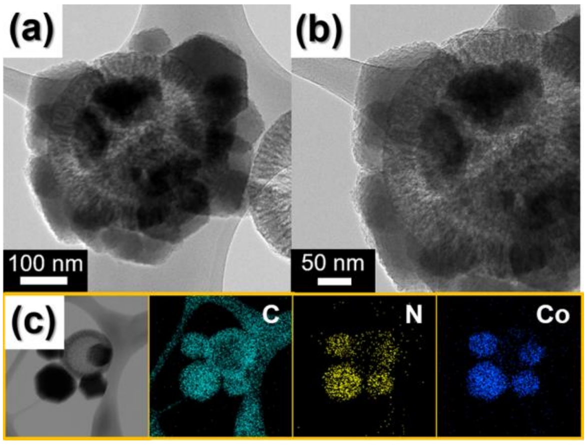

Fig. S6 Morphologies and elemental mapping images of ZIF-67/HMCS composite prepared under non-vacuum state: **a, b** TEM images and **c** elemental mapping images

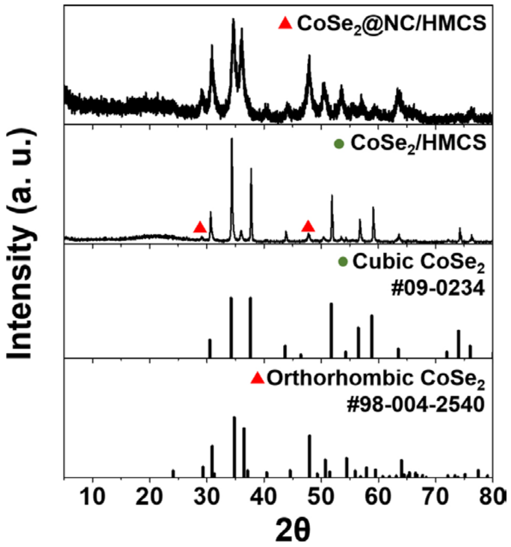

Fig. S7 XRD patterns of of $CoSe_2$@NC/HMCS and $CoSe_2$/HMCS composites

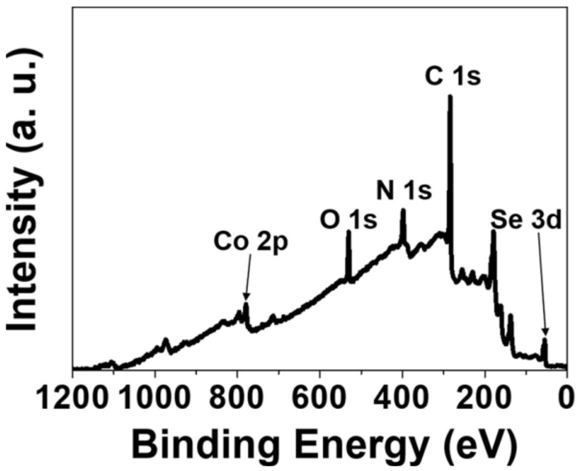

Fig. S8 XPS survey scan for $CoSe_2$@NC/HMCS composite

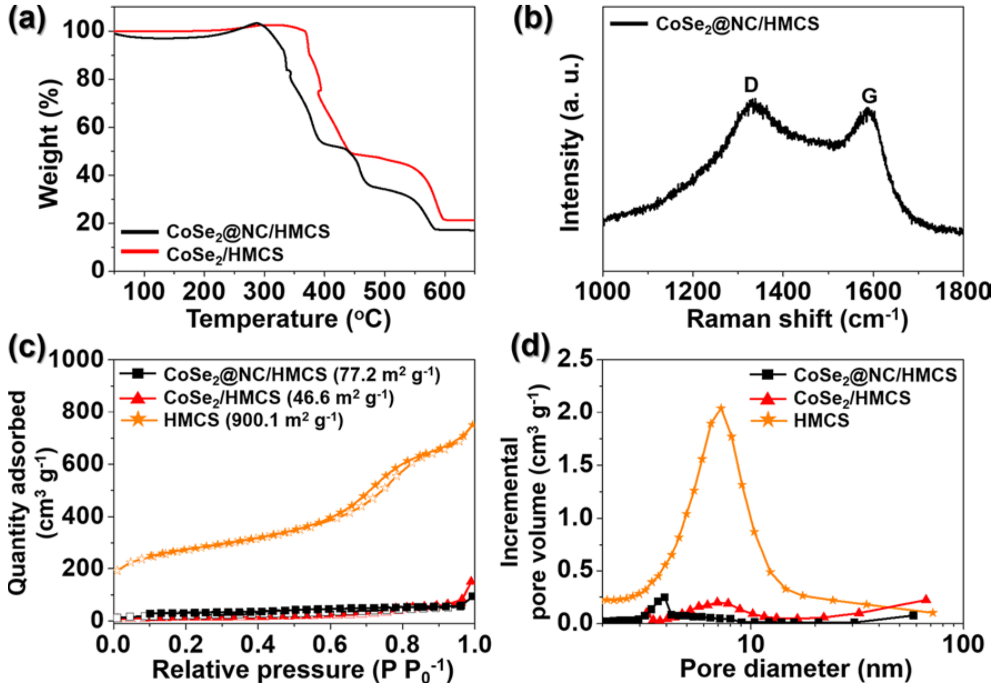

Fig. S9 **a** TG curves, **b** Raman spectra, **c** N_2 gas adsorption and desorption isotherms, and **d** BJH pore size distributions of HMCS, $CoSe_2$@NC/HMCS, and $CoSe_2$/HMCS composites

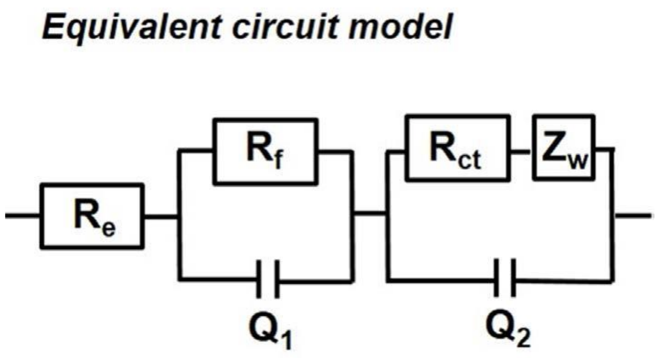

Fig. S10 Randle-type equivalent circuit model used for EIS fitting

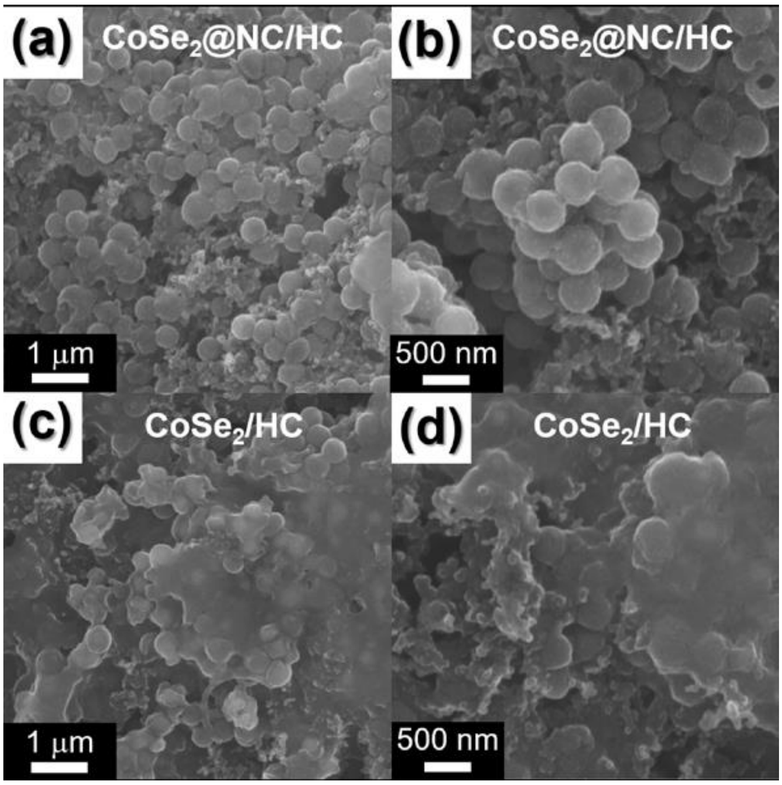

Fig. S11 Morphologies of **a, b** $CoSe_2$@NC/HMCS and **c, d** $CoSe_2$/HMCS composites after 100 cycles

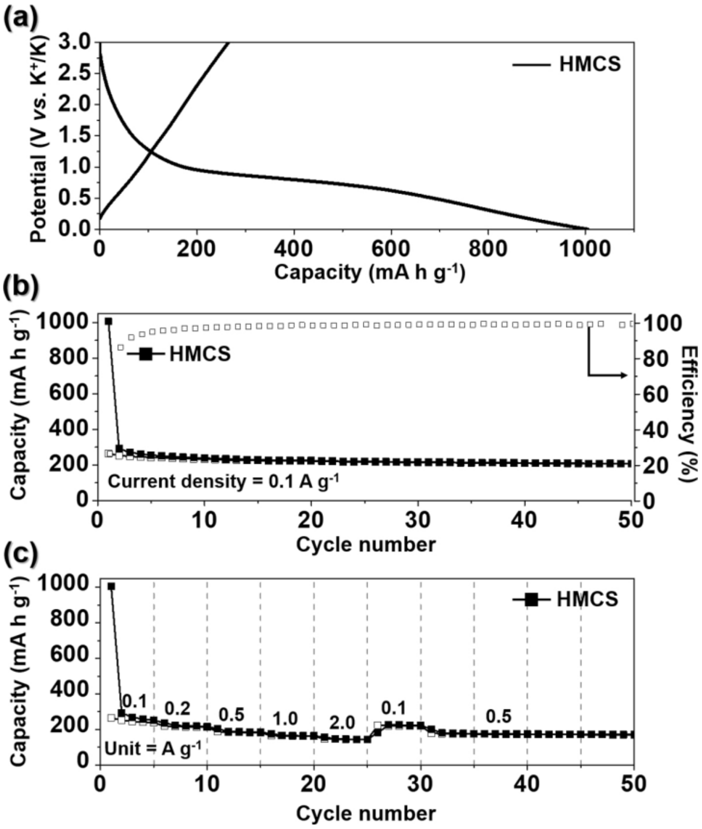

Fig. S12 Electrochemical properties of HMCS: **a** initial galvanostatic charge-discharge curves, **b** cycle performance at a current density of 0.1 A g^{-1}, and **c** rate performance at various current densities

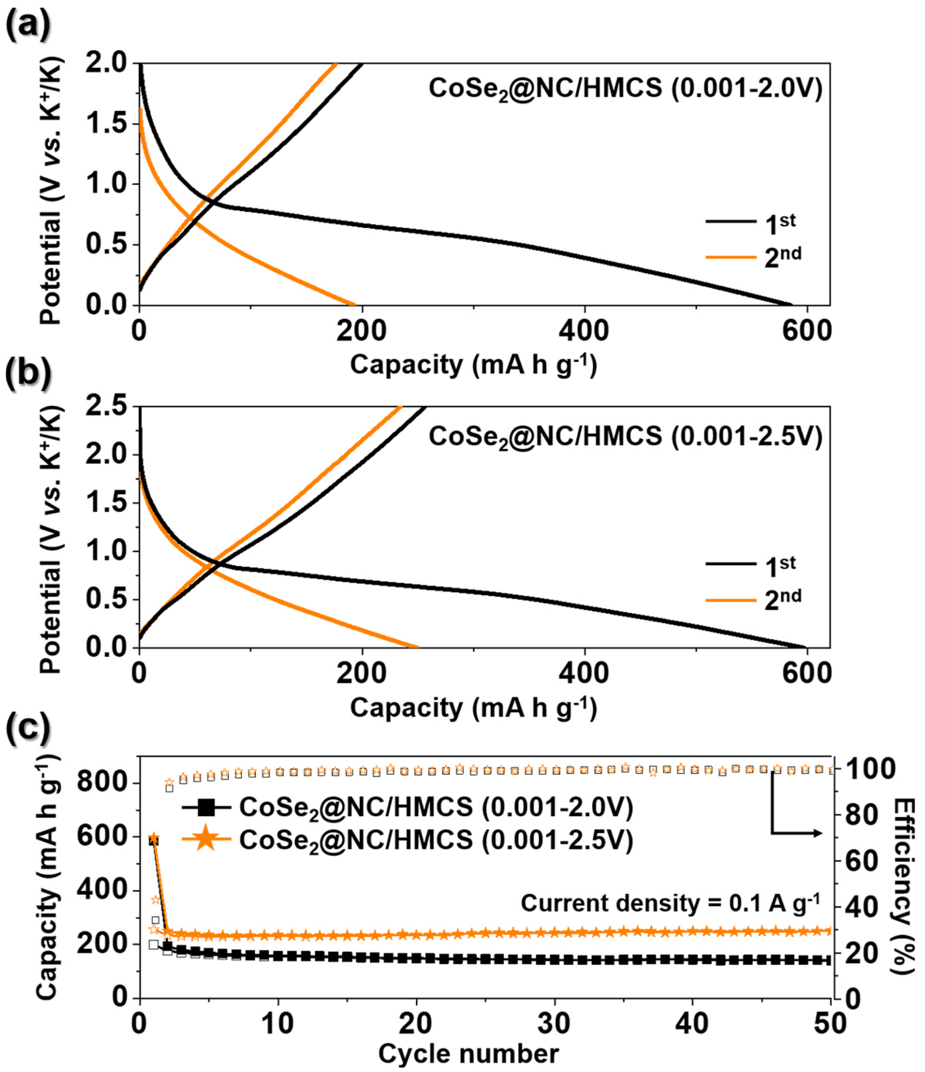

Fig. S13 Electrochemical properties of $CoSe_2$@NC/HMCS composite in the range of 0.001-2.0 and 0.001-2.5 V: **a, b** the first and second galvanostatic charge-discharge curves, **b** cycle performances at a current density of 0.1 A g^{-1}

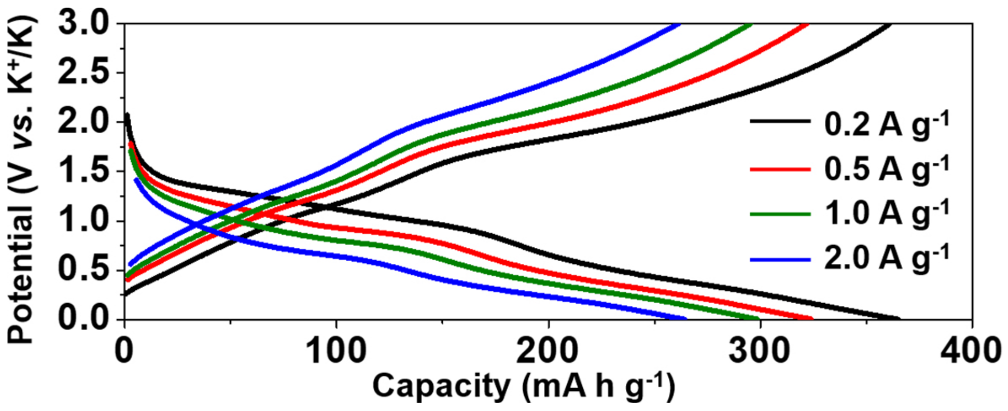

Fig. S14 Galvanostatic charge-discharge curves of $CoSe_2$@NC/HMCS composite at various current densities

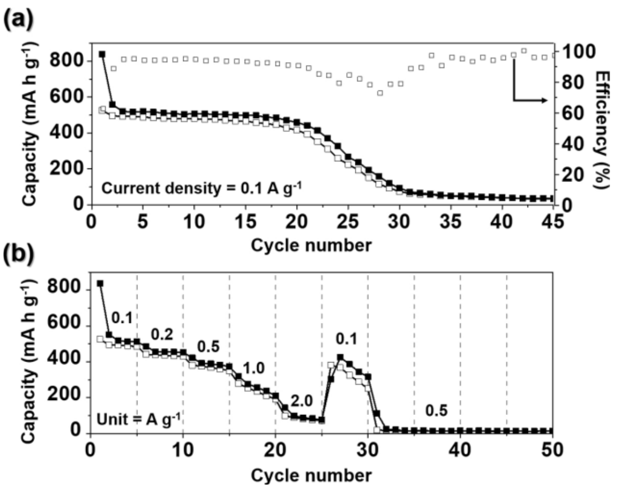

Fig. S15 Electrochemical properties of $CoSe_2$@NC/HMCS composite prepared under non-vacuum state: **a** cycle performance at a current density of 0.1 A g^{-1} and **b** rate performance at various current densities

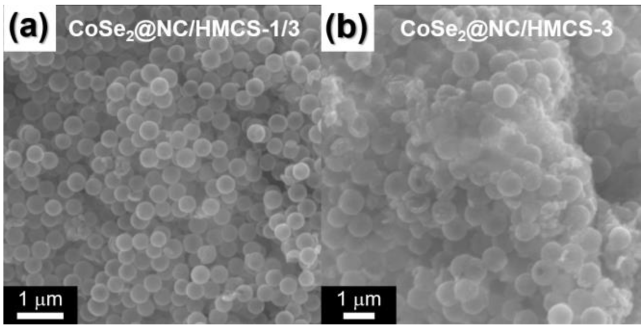

Fig. S16 Morphologies of $CoSe_2$@NC/HMCS-1/3 and $CoSe_2$@NC/HMCS-3 composites: **a, b** SEM images

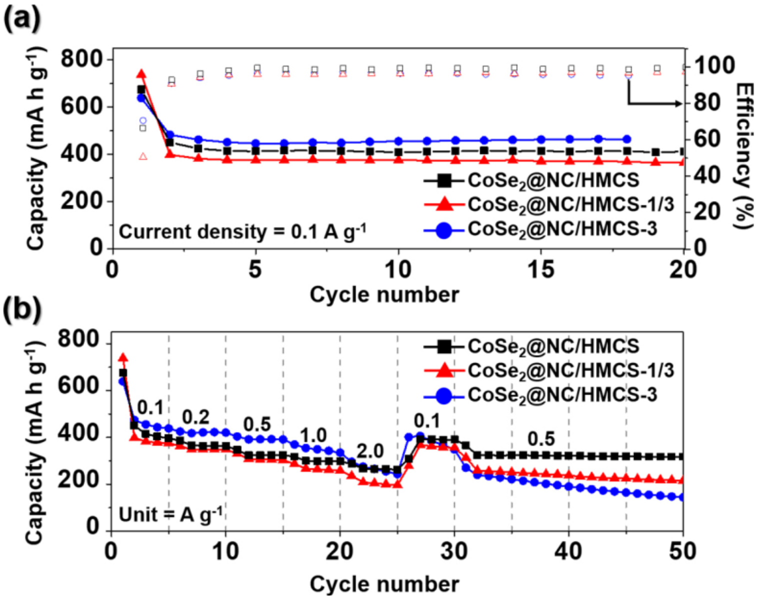

Fig. S17 Electrochemical properties of $CoSe_2$@NC/HMCS composites with different amount of Co-nitrate: **a** cycle performances at a current density of 0.1 A g^{-1}, and **b** rate performances at various current densities

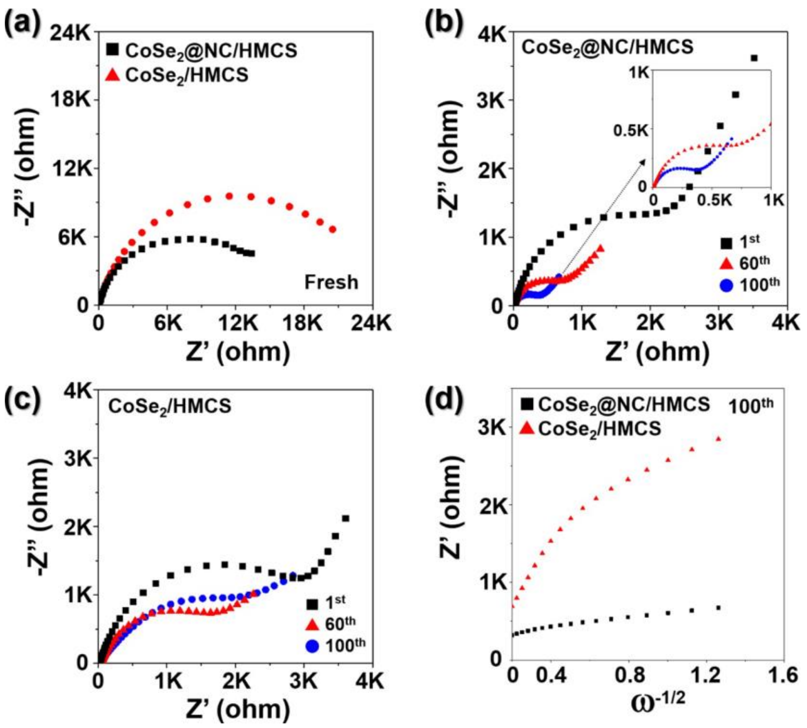

Fig. S18 Nyquist plots of **a** fresh cells, **b** after the 1st, 60th, and 100th cycle of $CoSe_2$@NC/HMCS composite, **c** after the 1st, 60th, and 100th cycle of $CoSe_2$/HMCS composite, and **d** the relationship between the phase angle ($\omega^{-1/2}$) and impedance (Z') of the two electrodes at the 100th cycle

Table S1 Electrochemical properties of various metal selenides materials applied as potassium-ion batteries reported in the previous literatures

Material	Voltage range (V)	Current rate (mA g^{-1})	Discharge capacity (mAh g^{-1})	Cycle number	Rate capacity (mAh g^{-1})	Refs.
**$CoSe_2$@NC/HMCS**	**0.001-3.0**	**100**	**442**	**120**	**263 (2.0 A g^{-1})**	**Our work**
N-doped carbon/ultrathin 2D metallic cobalt selenide	0.01-2.6	50	335	200	226 (2.0 A g^{-1})	[S1]
$Co_{0.85}Se$@C in carbon nanofibers film	0.01-2.6	200	353	100	166 (5.0 A g^{-1})	[S2]
$Co_{0.85}Se$ nanparticles in N-doped carbon	0.01-3.0	100	287	60	111 (2.0 A g^{-1})	[S3]
$CoSe_2$ threaded by N-doped carbon nanotubes	0.01-2.5	200	253	100	196 (2.0 A g^{-1})	[S4]
N-rich Cu_2Se/C nanowires	0.1-2.5	100	190	200	104 (2.0 A g^{-1})	[S5]
N-doped carbon-encapsulated ZnSe@C	0.01-3.0	200	360	60	168 (4.0 A g^{-1})	[S6]

$Co_{0.85}Se$ cubes encapsulated in graphene	0.01-2.6	50	402	200	260 (1.0 A g^{-1})	[S7]
$MoSe_2$/C nanostructures	0.01-2.5	200	322	100	224 (2.0 A g^{-1})	[S8]
$Co_{0.85}Se$ quantum dots/C composite	0.01-2.5	50	402	100	220 (2.0 A g^{-1})	[S9]

Supplementary References

[S1] G. Suo, J. Zhang, D. Li, Q. Yu, W.A. Wang et al., N-doped carbon/ultrathin 2D metallic cobalt selenide core/sheath flexible framework bridged by chemical bonds for high-performance potassium storage. Chem. Eng. J. **388**, 124396 (2020). https://doi.org/10.1016/j.cej.2020.124396

[S2] C.A. Etogo, H. Huang, H. Hong, G. Liu, L. Zhang, Metal–organic-frameworks-engaged formation of $Co_{0.85}Se$@C nanoboxes embedded in carbon nanofibers film for enhanced potassium-ion storage. Energy Storage Mater. **24**, 167-176 (2020). https://doi.org/10.1016/j.ensm.2019.08.022

[S3] G. Ma, C. Li, F. Liu, M.K. Majeed, Z. Feng et al., Metal-organic framework-derived Co0.85Se nanoparticles in N-doped carbon as a high-rate and long-lifespan anode material for potassium ion batteries. Mater. Today Energy **10**, 241-248 (2018). https://doi.org/10.1016/j.mtener.2018.09.013

[S4] Q. Yu, B. Jiang, J. Hu, C.Y. Lao, Y. Gao et al., Metallic octahedral $CoSe_2$ threaded by N-doped carbon nanotubes: a flexible framework for high-performance potassium-ion batteries. Adv. Sci. **5**(10), 1800782 (2018). https://doi.org/10.1002/advs.201800782

[S5] X. Zhu, J. Gao, J. Li, G. Hu, J. Li et al., Self-supporting N-rich Cu_2Se/C nanowires for highly reversible, long-life potassium-ion storage. Sustain. Energy Fuels **4**(5), 2453-2461 (2020). https://doi.org/10.1039/D0SE00160K

[S6] X. Xu, B. Mai, Z. Liu, S. Ji, R. Hu et al., Self-sacrificial template-directed ZnSe@C as high performance anode for potassium-ion batteries. Chem. Eng. J. **387**, 124061 (2020). https://doi.org/10.1016/j.cej.2020.124061

[S7] D. Li, J. Zhang, G. Suo, Q. Yu, W. Wang et al., Hollow $Co_{0.85}Se$ cubes encapsulated in graphene for enhanced potassium storage. J. Electroanal. Chem. **864**, 114100 (2020). https://doi.org/10.1016/j.jelechem.2020.114100

[S8] W. Wang, B. Jiang, C. Qian, F. Lv, J. Feng et al., Pistachio-shuck-like $MoSe_2$/C core/shell nanostructures for high-performance potassium-ion storage. Adv. Mater. **30**(30), 1801812 (2018). https://doi.org/10.1002/adma.201801812

[S9] Z. Liu, K. Han, P. Li, W. Wang, D. He et al., Tuning metallic Co0.85Se quantum dots/carbon hollow polyhedrons with tertiary hierarchical structure for high-performance potassium ion batteries. Nano-Micro Lett. **11**, 96 (2019). https://doi.org/10.1007/s40820-019-0326-5
